# The limits of market-based reforms

**DOI:** 10.1186/1472-6963-13-S1-I1

**Published:** 2013-05-24

**Authors:** Helen Dickinson, Sara Shaw, Jon Glasby, Judith Smith

**Affiliations:** 1 Health Services Management Centre, University of Birmingham, Birmingham, UK; 2 Queen Mary University London, London, UK; 3 The Nuffield Trust, London, UK

## Introduction

Every health and social care system around the world is concerned with how it can provide care in a way that ensures both high quality and cost-effective care for people. The English system is no exception and over the last few decades there has been increased interest in the use of markets within the context of health and social care [1]. Although local government in the UK has a longer track record of developing a market for care services (especially since the passage of the 1990 NHS and Community Care Act), a series of NHS reforms have followed suit, also drawing on market-based approaches (see [2,3]).

The initial introduction of an internal market into the English NHS in the 1990s saw the separation of the functions of service delivery (provision) and the purchasing and planning of these (commissioning). The context has since developed and today commissioners are being asked to strengthen the market further and enable the diversification of provision in a mixed economy of care. The recent passage of the Health and Social Care Act 2012 requires commissioners to ensure good practice and promote and protect patient choice. The legislation seeks to make the NHS more responsive, efficient and accountable by putting clinicians at the forefront of commissioning and, most controversially, opening up the field of provision to encourage more entrants to the market and, ultimately, greater competition [4,5]. Thus, market-based competition is one of the main tools that are available to health care commissioners working within the NHS in England. These changes echo those which are going on in other parts of the globe as national governments seek to develop and extend markets in health and social care.

Market based reforms have a central role within the English government’s plans for health and social care, however; they have not been warmly welcomed by all (to put it mildly). There has been considerable critique of the introduction and expansion of market-based reforms in health and social care [e.g. [6-8]]. Most recently there has been considerable debate about the Health and Social Care Act, generally and specifically in terms of the Section 75 regulations which on the face of it are concerned with ‘good procurement practice’, but which many feel open the NHS up to competition.

Against this background the Health Services Management Centre at the University of Birmingham and the Nuffield Trust jointly convened a seminar to explore the ‘limits of market-based reforms’ in health and social care and the papers that comprise this supplement are a product of the presentations, discussions and debates from that day. Questions addressed during the day included:

• What are the theories that have underpinned the study of commissioning?

• What are the ways in which we can investigate commissioning?

• What does it mean to do commissioning?

• What is the evidence base for commissioning?

• What is the future of commissioning?

Ultimately, this seminar was particularly concerned with understanding what we know about the effectiveness of market-based reforms and from a variety of disciplinary and methodological perspectives.

## Overview of supplement

This supplement opens with Pauline Allen’s paper [9], which reviews the literature on the fundamentals of markets and examines the application of market concepts to the delivery of health care. Drawing on this literature and evidence from the NHS, Allen argues that there are serious limitations to the efficacy of using economic market principles in the delivery of healthcare. The paper provides an introduction to the key concepts relating to markets and their application in health care and is a foundation upon which subsequent papers build.

Having set out this background, we then move on to a case study in the English NHS, with Alison Porter and colleagues’ paper on commissioning healthcare for people with long term conditions [10]. Porter et al examine the extent to which local commissioners have adopted a market-oriented model of commissioning of care for people with long term conditions. They conclude that despite the rhetoric of market based reform and the emphasis on using transactional mechanisms such as contracts, in practice commissioners in the NHS tend to operate in a more relational way with providers, based on trust and collaboration. None of the six service areas they examined in their research exhibited the characteristics of a well functioning quasi-market.

The next two papers focus on commissioning from non-traditional providers in the English NHS. Anna Coleman and colleagues investigate commissioning Alternative Providers of Primary Care (APPCs) as a means of delivering primary care services to meet local needs, but outside of traditional general medical practices [11]. This study finds that APPCs struggled to build up their list sizes whilst over performing on other aspects of their contracts such as walk-in numbers. The form of contracting used in these cases tended to be transactional in nature as opposed to the relational contracting typically employed in the NHS. As such the process of contracting tended to be costly, although this competition amongst providers had led some practices to improve their services. Ultimately the authors conclude that if a market is to operate in primary care in the English NHS then there is the need for a serious debate about potential trade-offs between factors such as cost, performance assurance, transparency and fair procurement processes.

Following Coleman et al, Naomi Chambers and colleagues describe a case study of commissioning which focuses on a partnership between the NHS and a private provider [12]. In this case the private provider acted as co-commissioner and provider – alongside an NHS general practice – to redesign local primary care services and support the commissioning of services for people with long term conditions at risk of unplanned hospital admissions. Chambers et al describe a very close relationship between commissioner and provider based on relational ways of working, and in contrast to more transactional aspects observed by Coleman et al. The findings of Chambers et al align with those of Alison Porter and colleagues, suggesting, perhaps, that the process of planning and providing long term condition services requires these sorts of relationships.

The next four papers take a broader focus on commissioning, the first two consider commissioning beyond the remit of the NHS and the second two provide a comparison of the English NHS with other national contexts.

Catherine Needham explores personal budgets in social care services and the limitations of the market in relation to individual purchasers of private goods, the pooling of funds to purchase group services and in the provision of public goods [13]. Ultimately Needham finds serious challenges within the English experience, where the accompanying financial crisis has led to an undersupply of collective and public goods. The lack of service availability challenges the idea of consumers making active choices about care from a range of providers in the marketplace.

Helen Dickinson and colleagues then consider the issue of joint commissioning between health and social care [14]. While this has been firmly advocated in national policy, Dickinson et al demonstrate that there is little evidence to demonstrate that joint commissioning improves outcomes for services users. This paper investigates in detail the claims that are made about joint commissioning within the literature and identifies at least three discourses – empowerment, prevention and efficiency – each of which suggests that joint commissioning is attempting to achieve something slightly different. Dickinson et al conclude that what is of interest is how local organisations go about attempting to implement joint commissioning within the context of such conceptual ambiguity.

Next, David Hughes and colleagues examine secondary care contracting in England and Wales during a period of increasing divergence between the two systems [15]. Hughes et al found that even though the policy systems for these two countries were increasingly different with England making greater use of market mechanisms, in practice long-term relationships between partners had an important part to play in both. Both systems had elements of cooperation and conflict and, in practice, looked more similar than their policy contexts would suggest.

Finally, Rod Sheaff and colleagues examine commissioning as governance, contrasting approaches in two different national settings of England and Germany and examine the methods of control that each employ [16]. This study finds that even where the same control mechanisms are used to steer the systems of these countries it may take a different form depending on the other controls that co-exist and the national political cultures. Ultimately Sheaff and colleagues conclude that there are limits to market based reforms in both countries as different forms of power act to frustrate one another in practice.

Overall these papers demonstrate that, despite the rhetoric about the operation of a market in health, there are limits to this both theoretically and empirically. As Allen demonstrates, market based reforms are theoretically flawed and unable to operate in the way intended of markets. This is also illustrated through studies of the operation of commissioning in the NHS (Porter et al, Chambers et al, Hughes et al and Sheaff et al), where there is little, if any, evidence of a market functioning in practice. Even where a market is operating in a transactional way as Coleman et al demonstrate, there are questions about whether this is desirable when the potential benefits are weighed against other factors such as reduced transparency or performance. From these studies it would appear that beyond central government direction about what markets should deliver, the interpretations ‘on the ground’ are often different. Many of the papers illustrate the power of health professionals in particular to guard against the worst elements of market reform and enable continuity through collaboration with longstanding partners. However, there is a warning message in Needham’s paper from the context of social care where the personalisation agenda combined with austerity measures have eroded the ability of professionals to carry on despite the system.

Taken together the papers presented in this supplement clearly raise questions about the future of reforms in England – and across the world – that attempt to introduce and embed markets in health and social care. Whilst they offer diversity in terms of their theoretical, disciplinary and/or empirical focus, they offer remarkably similar conclusions about the limited potential of markets in health and social care to deliver aspirations for improvements in both the quality and cost of care. Those involved in developing and attempting to deliver market-based reforms would do well to pay heed to the concerns that authors raise.

## Declarations

Publication of this supplement was funded by the Nuffield Trust.

This article has been published as part of *BMC Health Services Research* Volume 13 Supplement 1, 2013: The Limits of Market-based Reforms. The full contents of the supplement are available online at http://www.biomedcentral.com/bmchealthservres/supplements/13/S1.
